# Satisfaction and adherence to pharmacological treatment for glycemic control in adults

**DOI:** 10.15649/cuidarte.4128

**Published:** 2025-11-24

**Authors:** Jessica Jazmín Leyva-Perea, Patricia Enedina Miranda-Félix, Juana Mercedes Gutiérrez-Valverde, Rosario Edith Ortiz-Félix, Luis Antonio Mancillas-Berrelleza

**Affiliations:** 1 Master of Science in Nursing, Mochis School of Nursing of the Universidad Autónoma de Sinaloa. Los Mochis, Sinaloa, Mexico. E-mail: jessijleyva20@gmail.com Mochis School of Nursing of the Universidad Autónoma de Sinaloa Sinaloa Mexico jessijleyva20@gmail.com; 2 Master of Science in Nursing, Mochis School of Nursing of the Universidad Autónoma de Sinaloa. Los Mochis, Sinaloa, Mexico. E-mail: patriciamiranda@uas.edu.mx Mochis School of Nursing of the Universidad Autónoma de Sinaloa Sinaloa Mexico patriciamiranda@uas.edu.mx; 3 Professor and Researcher of the School of Nursing, Universidad Autónoma de Nuevo León. Monterrey, Nuevo León, Mexico. E-mail: juana.gutierrezvl@uanl.edu.mx Universidad Autónoma de Nuevo León Nuevo León Mexico juana.gutierrezvl@uanl.edu.mx; 4 Professor and Researcher at the Mochis School of Nursing of the Universidad Autónoma de Sinaloa. Los Mochis, Sinaloa, Mexico. E-mail: rosarioortiz@uas.edu.mx Mochis School of Nursing of the Universidad Autónoma de Sinaloa Sinaloa Mexico rosarioortiz@uas.edu.mx; 5 Master of Science in Nursing, Facultad de Enfermería Mochis de la Universidad Autónoma de Sinaloa; Hospital IMSS Bienestar del Bajo Río Mayo. Huatabampo, Sonora, Mexico. E-mail:luanmabe0103@gmail.com Universidad Autónoma de Sinaloa; Hospital IMSS Bienestar del Bajo Río Mayo Sonora Mexico luanmabe0103@gmail.com

**Keywords:** Type 2 Diabetes, Medication Adherence, Glycemic Control, Patient Satisfaction, Diabetes Tipo 2, Adhesión al Tratamiento Farmacológico, Control Glucémico, Satisfacción del Paciente, Diabetes tipo 2, Adesão ao Tratamento Medicamentoso, Controle Glicêmico, Satisfação do Paciente

## Abstract

**Introduction:**

The management of Type 2 Diabetes should be based on a person-centered approach to ensure the appropriate selection of pharmacological treatment. Satisfaction with pharmacological treatment is a patient-reported assessment measure that evaluates the treatment process and related factors, which may enhance adherence and glycemic control.

**Objective:**

To determine the strength of association between satisfaction with pharmacological treatment and adherence for glycemic control in adults with Type 2 Diabetes.

**Materials and Methods:**

A cross-sectional predictive study with model testing was conducted. A total of 146 participants with Type 2 Diabetes were enrolled using snowball sampling. The Diabetes Treatment Satisfaction Questionnaire (DTSQ), the Morisky Medication Adherence Scale (MMAS-4), and a sociodemographic and clinical data form were administered, and glycated hemoglobin level was measured. Associations were estimated using multiple linear regression.

**Results:**

Adherence to pharmacological treatment only predicted glucose control. The difference in glycated hemoglobin between adherent and non-adherent individuals was 0.22 lower in adherent individuals (β = -0.22, F = 2.95, p = 0.001).

**Discussion:**

The results differ from other studies that have reported subsequent changes in glycated hemoglobin associated with treatment satisfaction.

**Conclusion:**

Adherence to pharmacological treatment influences glycemic control. It is essential for healthcare professionals to consider this evidence for decision- making and in the design, implementation, and/or reinforcement of educational interventions.

## Resumen

**Introducción:**

El manejo de la Diabetes Tipo 2 debe ser con un enfoque centrado en la persona para la selección apropiada del tratamiento farmacológico. La satisfacción con el tratamiento farmacológico es una medida de evaluación referida por la persona en donde se evalúa el proceso del tratamiento y factores relacionados con él, lo cual puede favorecer su adherencia y el control glucémico.

**Objetivo:**

Determinar la fuerza de asociación de la satisfacción con el tratamiento farmacológico y su adherencia sobre el control glucémico en adultos con Diabetes Tipo 2.

**Materiales y métodos:**

Predictivo transversal con comprobación de modelo. Fueron 146 participantes con Diabetes Tipo 2, muestreo por bola de nieve. Se aplicaron los cuestionarios Diabetes Treatment Satisfaction Questionnaire, Morisky Medication Adherence Scale MMAS-4, cédula de datos sociodemográficos y clínicos, medición de Hemoglobina Glucosilada. La asociación se estimó mediante regresión lineal múltiple.

**Resultados:**

La adherencia al tratamiento farmacológico sólo predijo el control de la glucosa, la diferencia de Hemoglobina Glucosilada entre las personas con adherencia al tratamiento versus las no adherentes es 0,22 menor (β= -0,22, F =2,95, p = 0,001).

**Discusión:**

Los resultados difieren de otros estudios en cuanto a la satisfacción con el tratamiento quienes han referido cambios posteriores en Hemoglobina Glucosilada.

**Conclusiones:**

La adherencia al tratamiento farmacológico influye en el control glucémico, es esencial que el personal de salud conozca esos datos para la toma de decisiones, diseño e implementación y/o reforzamiento de intervenciones educativas.

## Resumo

**Introdução:**

O manejo do diabetes tipo 2 deve adotar uma abordagem centrada na pessoa para a seleção adequada do tratamento farmacológico. A satisfação com o tratamento farmacológico é uma medida de avaliação autorrelatada que avalia o processo de tratamento e os fatores relacionados, que podem promover a adesão e o controle glicêmico.

**Objetivo:**

Determinar a força da associação entre a satisfação com o tratamento farmacológico e a adesão ao controle glicêmico em adultos com diabetes tipo 2.

**Materiais e métodos:**

Modelo preditivo transversal com teste de modelo. Foram incluídos 146 participantes com diabetes tipo 2, amostragem por bola de neve. Foram aplicados o Questionário de Satisfação com o Tratamento do Diabetes, a Escala de Adesão à Medicação de Morisky (MMAS- 4), uma coleta de dados sociodemográficos e clínicos e uma dosagem de hemoglobina glicada. A associação foi estimada por meio de regressão linear múltipla.

**Resultados:**

A adesão ao tratamento medicamentoso apenas previu o controle glicêmico; a diferença na hemoglobina glicada entre pessoas com adesão ao tratamento e não aderentes foi 0,22 menor (β = -0,22, F = 2,95, p = 0,001).

**Discussão:**

Os resultados diferem de outros estudos quanto à satisfação com o tratamento, que relataram alterações subsequentes na hemoglobina glicada.

**Conclusões:**

A adesão ao tratamento medicamentoso influencia o controle glicêmico; é essencial que os profissionais de saúde conheçam esses dados para a tomada de decisões, o planejamento e a implementação e/ou o reforço de intervenções educacionais.

## Introduction

Type 2 Diabetes (T2D) is a chronic disease that occurs when insulin is not used effectively or is insufficient, causing the accumulation of glucose in the blood^1^. Worldwide, 588.7 million adults aged 20 to 79 live with diabetes, making it one of the top 10 causes of death; the increase in these figures is mainly attributed to T2D, which represents more than 90% of diabetes cases and is due to socioeconomic, demographic, environmental, and genetic factors^2^. According to the most recent data from the 2022 National Health and Nutrition Survey (ENSANUT), the prevalence of T2D among adults in Mexico was 18.3%^3^, and 10.7% in the state of Sinaloa in 2018^4^.

The primary goal of the health system following diagnosis is to ensure T2D control^5^. Poor glycemic control favors the development of long-term complications; therefore, carrying out actions such as using pharmacological medications is necessary^6^. Due to the high prevalence of T2D, educational strategies have been established to promote behavioral change to control the disease; however, a high number of adults still remain uncontrolled^7^.

In Mexico, 68.2% of the population diagnosed with T2D exhibit uncontrolled blood glucose, that is, glycated hemoglobin (HbA1c) levels ≥ 7%, according to the parameters established by the American Diabetes Association (ADA); this condition increases the risk of complications at an earlier age^5^. The ADA establishes that the management of T2D should be guided by a person-centered approach to ensure appropriate pharmacological therapy selection^8^. Satisfaction with pharmacological treatment is an evaluation measure of the treatment process and related factors reported by the patient^9^.

Due to the need for control, it has been demonstrated that satisfaction with the pharmacological treatment of adults with T2D should be considered, as this could improve adherence to treatment, thereby improving glycemic control and clinical outcomes^10^. Several studies have shown that treatment satisfaction has been independently associated with patients' adherence to the treatment and adequate hemoglobin levels^11^,^12^.

It is estimated that around 50% of people with chronic diseases adhere to treatments, but this may vary depending on the disease^13^. Optimal adherence to medical instructions by individuals may be compromised by barriers related to treatment characteristics, disease-specific factors, and an individual's contextual characteristics^7^. Adherence to pharmacological treatment is one of the actions that must be carried out to achieve disease control, as it contributes to the control of blood glucose levels^14^. The objective of this study was to determine the strength of the association between satisfaction with pharmacological treatment, adherence, and glycemic control in adults with T2D.

## Materials and Methods

**Study design and participants**

A cross-sectional predictive study with model testing was conducted to determine the strength of association between satisfaction with pharmacological treatment and adherence for glycemic control in adults with T2D. This study involved 146 adults with a previous diagnosis of T2D, aged between 18 and 70 years, both sexes, living in communities in Sinaloa (Ahome, El Fuerte and Guasave). Those adults who attended a health institution for medical check-ups and were receiving oral and/or injected pharmacological treatment were included.

**Variables of interest**

To measure the characteristics of the participants, a sociodemographic data form was used, including sex, age, marital status, ethnicity, education, socioeconomic level, and occupation. Clinical data included time since diagnosis, type of pharmacological treatment, changes of treatment, duration of treatment, and comorbidities.

Cut-off points for some variables were established taking into account criteria used in previous studies to classify the population and facilitate the analysis of differences between variables^7^.

Satisfaction with pharmacological treatment. In order to measure treatment satisfaction, the Spanish version of the Diabetes Treatment Satisfaction Questionnaire (DTSQ) validated by Gomis 2006^15^ was used. Designed for patients with T2D treated with oral hypoglycemic agents, insulin, and/or diet, the DTSQ consists of 8 items scored on a Likert-type scale ranging from 0 (very dissatisfied) to 6 (extremely satisfied). The sum of 6 of the 8 items yields an overall satisfaction score ranging from 0 points (lowest possible satisfaction) to 36 points (highest possible satisfaction). Additionally, the overall score was classified into three categories: 0-11, very dissatisfied; 12-23, moderately satisfied; and 24-36, very satisfied. The remaining two items, which refer to episodes of perceived hyperglycemia and hypoglycemia, were individually and descriptively analyzed. The DTSQ has been validated in the Spanish population, was endorsed by the WHO and the International Diabetes Federation, and reported a Cronbach’s alpha of 0.907.

Adherence to pharmacological treatment. Adherence was measured using the Morisky Medication Adherence Scale (MMAS-4)^16^, validated in the Mexican population. It consists of 4 items with dichotomous (YES/NO) responses to questions such as: Do you ever forget to take your medication? Patients are considered adherent to treatment if they answer the questions correctly, i.e., NO/YES/ NO/NO. The scale reported a Cronbach’s alpha of 0.67.

Glycemic control. In order to determine glycemic control, Hb1Ac <7% was defined as a biochemical indicator in accordance with ADA glycemic targets^17^. The compact ApexBio® Eclipse A1C POC analyzer (Health Registration No. 0123E2017 SSA; certified by the National Glycohemoglobin Standardization Protocol in 2020) was used.

**Statistical analysis**

The Statistical Package for the Social Sciences (SPSS) version 26 was used. Descriptive statistics (frequencies, percentages, means, and standard deviation) were used to describe sociodemographic characteristics. The Kolmogorov-Smirnov (K-S) test was used to assess data distribution, which was found to be non-parametric (p <0.05). Inferential analyses were conducted to determine differences between sociodemographic/clinical variables and glycemic control using the Mann-Whitney U and Kruskal-Wallis tests. A multiple regression model was subsequently run using the enter method with continuous and ordinal variables, and those that showed statistical significance (p <0.05). The study data are available in Mendeley Data^18^.

**Ethical considerations**

This study adhered to the regulations of the General Health Law on Research^19^. Likewise, it met the provisions of the Mexican Official Standard NOM-087-ECOL-SSA1-2002 on Environmental Protection - Environmental Health - Biological-Infectious Hazardous Waste. Approval was granted by the Ethics and Research Committee of the Mochis Faculty of Nursing of the Universidad Autónoma de Sinaloa (Registration number CONBIOETICA-25-CEI-001-20211201).

## Results

 The sample consisted of 146 participants diagnosed with T2D. Table 1 shows the sociodemographic and clinical characteristics of the participants. Of the total sample, 69.86% (n = 102) were women and 30.14% (n = 44) were men. The mean age of the participants was 55.51 years (SD = 9.83), and 89.04% (n = 130) had ≥ 5 years of education. Regarding clinical data, 58.22% (n = 85) of the participants had been diagnosed with T2D for 5 years or more, 56.85% (n = 83) used oral medications, and 58.22% (n= 85) reported having associated comorbidities. The mean HbA1c level was 7.72 (SD= 2.06).

 In the description of the variables of interest, 93.84% (n = 137) of the total sample reported being very satisfied with their current pharmacological treatment. It was found that 58.22% (n = 85) did not adhere to treatment and 50.68% (n = 74) had poor glycemic control, defined as HbA1c > 7% according to ADA glycemic targets. In addition, differences were found between some variables and glycemic control, such as type of treatment (p = .001), change of treatment (p = 0.001), and duration of treatment (p = 0.001). Specifically, adults receiving oral treatment had better glycemic control than those using injectable treatment or a combination of both. Regarding treatment changes, those who reported no changes in the type of treatment had better glycemic control. Similarly, adults who reported having been on treatment for less than 5 years had greater glycemic control (See Table 1).

**Table 1:** Description of sociodemographic/clinical characteristics, comparison, and differences in glycemic control

Variables	Total %(n) (146)	Good Control %(n) (72)	Poor Control %(n) (74)	p-value
Sex				0.640+
Woman	69.86 (102)	68.06 (49)	71.62 (53)	
Man	30.14 (44)	31.94 (23)	28.38 (21)	
Age				0.936+
>65 years	19.18 (28)	19.44 (14)	18.92 (14)	
<65 years	80.82 (118)	80.56 (58)	81.08 (60)	
Marital status				0.246+
With partner	70.55 (103)	75.00 (54)	66.22 (49)	
Without partner	29.45 (43)	25.00 (18)	33.78 (25)	
Socioeconomic status				0.737++
Lower class	24.66 (36)	25.00 (18)	24.32 (18)	
Low class	63.70 (93)	61.11 (44)	66.22 (49)	
Lower-middle class	11.64 (17)	13.89 (10)	9.46 (7)	
Middle class	0.00 (0)	0.00 (0)	0.00 (0)	
Upper-middle class	0.00 (0)	0.00 (0)	0.00 (0)	
Upper class	0.00 (0)	0.00 (0)	0.00 (0)	
Education				0.954+
>5 years	89.04 (130)	88.89 (64)	89.19 (66)	
<5 years	10.96 (16)	11.11 (8)	10.81 (8)	
Occupation				0.032+
Paid	49.32 (72)	58.33 (42)	40.54 (30)	
Unpaid	50.68 (74)	41.67 (30)	59.46 (44)	
Years since DT2 Dx				0.001+
>5 years	58.22 (85)	43.06 (31)	72.97 (54)	
<5 years	41.78 (61)	56.94 (41)	27.03 (20)	
Type of treatment				0.001++
Oral	56.85 (83)	75.00 (54)	39.19 (29)	
Injectable	6.85 (10)	1.39 (1)	12.16 (9)	
Both	36.30 (53)	23.61 (17)	48.65 (36)	
Change of treatment				0.001+
Yes	39.73 (58)	20.83 (15)	58.11(43)	
No	60.27 (88)	79.17 (57)	41.89 (31)	
Duration of treatment				0.008+
>5 years	45.89 (67)	34.72 (25)	56.76 (42)	
<5 years	54.11 (79)	65.28 (47)	43.24 (32)	
Comorbidities				0.759+
Yes	58.22 (85)	59.46 (44)	56.94 (41)	
No	41.78 (61)	40.54 (30)	43.06 (31)	
Satisfaction with treatment				0.094++
Very dissatisfied	0 (0)	0 (0)	0 (0)	
Moderately satisfied	6.16 (9)	2.78 (2)	9.46 (7)	
Very satisfied	93.84 (137)	97.22 (70)	90.54 (67)	
Adherence to treatment				
Adherent	41.78 (61)	56.94 (41)	27.03 (20)	
Non-Adherent	58.22 (85)	43.06 (31)	72.97 (54)	
HbA1c				
Means ± SD	7.72 ± 2.06			
Median (Q1; Q3)	7.00 [6.40 ; 8.80]			
Range Min - Max	4.70 - 19.36			

Note: n = Number of participants, % = percentage, Dx = Diagnosis, DT2 = Type 2 Diabetes, HbA1c = Glycated hemoglobin, SD = Standard Deviation, Min = Minimum, Max = Maximum, + Mann-Whitney U test, ++ Kruskal-Wallis test.

Differences between study variables and years since T2D diagnosis were also estimated, showing that treatment adherence and glycemic control may vary as a result of disease duration (See Table 2).

Table 3 shows the results of a multiple linear regression model using the enter method. The model was statistically significant (β= -0.22, F =2.95, p = 0.001), with an explained variance of 15% (R2 = 0.15). The “very satisfied” category was excluded from the model. According to the model, adherence to pharmacological treatment was a significant predictor of glucose control. The glycated hemoglobin difference between individuals who reported adherence to treatment and those who do not adhere to treatment is .22 lower in those who adhere to treatment.

**Table 2:** Differences in treatment satisfaction, treatment adherence, and glycemic control by years since T2D Diagnosis

Variables	Years since the T2D diagnosis						
	< 5 years n=61		≥ 5 years n=85				
	Mean rank	Rank sum	Mean rank	Rank sum	U	Z	p-value
Satisfaction with treatment	78.00	4758.00	70.27	5973.00	2318.0	-2.61	0.090
Adherence to treatment	81.30	4959.00	67.91	5772.00	2117.0	-2.20	0.027*
Glycemic Control	86.57	5280.50	64.12	5450.50	1795.5	-3.65	0.001*

Note: n=146, DT2 = Type 2 Diabetes, U = Mann-Whitney U-test statistic, Z = standard deviation of the sum of ranks, *p-value <0,05.

**Table 3:** Multiple linear regression model for glycemic control

Variable	B	95% IC		SE B	β	t	p-value
		LL	UL				
Occupation	-0.110	-0.871	0.651	0.385	-0.027	-0.287	0.775
Years since diagnosis	-0.282	-1.432	0.869	0.582	-0.067	-0.484	0.629
Type of treatment	-0.015	-0.783	0.753	0.388	-0.007	-0.039	0.969
Duration of treatment	0.773	-0.260	1.806	0.522	-0.187	1.480	0.141
Changes in treatment	-1.440	-3.048	0.169	0.813	-0.342	-1.771	0.079
Satisfaction with Treatment							
Moderately satisfied	-0.560	-1.971	0.851	0.713	-0.065	-0.786	0.433
Adherence to treatment	-0.941	-1.634	-.248	0.350	-0.225**	-2.687	0.008**

Note: B= Unstandardized coefficient B, LL= Lower Limit, UL= Upper Limit, SE B= Stand Error of B, model explanation (R2 =0.240, adjusted R2 = 0.159). *p < 0.05 **p < 0.01.

## Discussion

The results obtained regarding sociodemographic characteristics are similar to those reported in other studies, showing a predominance of female participants. This may be explained by the higher prevalence of T2D among Mexican women^4^,^7^,^20^,^21^. Regarding years of education, the participants have more than five years, a result similar to that reported by Toledo et al.^7^ and Abu et al.^22^. These results also align with data from the National Institute of Statistics and Geography, which reports that among people aged 15 years and older, the average number of years of schooling is 9.7^23^. The predominant age group in the present study was comprised of individuals younger than 65 years. According to Statista’s research department, in 2019, worldwide, people with diabetes were predominantly between 20 and 64 years of age, and this number is expected to increase over time^24^.

Regarding clinical characteristics, participants reported having been diagnosed with T2D for more than five years, a result similar to that reported by other studies^20^-^22^,^25^. Oral pharmacological treatment was the most frequently reported therapy, which is consistent with ENSANUT data showing that 67.1% of adults with T2D use oral hypoglycemic agents^4^,^26^. This may be explained by the fact that oral pharmacological therapy is typically the first-line treatment, and many participants in this study reported not having experienced a change in their type of treatment.

In the present study, more than 50% of participants reported other comorbidities, similar to those reported by Guzmán et al.^27^, where more than 60% of participants reported at least one comorbidity. This result is expected, as comorbidities are common among people with T2D^28^.

Regarding the variables of interest (treatment satisfaction, adherence, and glycemic control), the results indicate that a high percentage of participants were moderately satisfied to very satisfied with their current treatment. Similar results were reported by Pascal and Nkwa^12^, Mancera-Romero et al.^10^, and Yaron et al.^29^. This finding could be attributed to the fact that most people perceive their current pharmacological treatment as flexible and practical and agree to continue and recommend it. Furthermore, some people reported that taking medication helps them control their disease and prevent complications^30^.

Regarding adherence to pharmacological treatment, participants reported a lack of adherence. This may be due to forgetfulness or discontinuation of therapy when participants felt better. These findings differ from those reported by Pascal and Nkwa^12^, who found in their study that participants did report adherence to treatment. Toledo et al. mentioned that only half of individuals with chronic illnesses adhere to treatment^7^. In this regard, the RedGDPS points out that people fail to take their medications because they do not follow medical prescriptions correctly^31^. Regarding glycemic control, more than half of the participating population exhibited poor glycemic control, which could be explained by non-adherence to treatment. This is consistent with Basto-Abreu et al.^5^, who reported that 68.2% of Mexican adults with T2D have poor glycemic control.

The analysis of differences revealed statistically significant associations, particularly regarding occupation, where differences were found in glycemic control. Participants who reported receiving a payment had better glycemic control. This may be explained by the fact that individuals with higher income are more likely to have access to health services and, consequently, to treatments^32^. Significant differences in glycemic control were also observed by type of pharmacological treatment, which may be attributed to the lower effectiveness and higher cost of injectable treatments. The findings of this study are consistent with previous studies showing that oral therapy is associated with greater glycemic control, as well as with the findings from another study in which adults using insulin had higher HbA1c levels^33^-^35^.

Regarding treatment changes, the results show differences, as participants who reported no changes in their treatment had better glycemic control. This may be attributed to the fact that most of these participants reported having been on treatment for fewer than five years, and they were initially prescribed oral treatment, which in turn has shown greater glycemic control compared to other types of treatment. There were significant differences in treatment adherence and glycemic control by years of diagnosis, which is consistent with Zhou et al.^20^, who reported that the duration of T2D was related to treatment adherence. This may be because treatment adherence and glycemic control can vary depending on the duration since T2D diagnosis. People with more years since diagnosis report lower adherence due to treatment complexity, while those with fewer years since diagnosis have less experience, as they have had less time with DT2^36^.

According to the regression model, only adherence to pharmacological treatment influences glycemic control; participants who reported adherence had better glycemic control. The findings are consistent with those reported by García et al.^21^, who indicate that adherence to treatment is associated with glycemic control. The results may be explained by the fact that adherence to pharmacological treatment is considered a protective factor for people's glycemic control^34^.

Regarding satisfaction with treatment, the findings show that it does not influence glycemic control. This result differs from that reported by Yaron et al.^29^, who found that satisfaction with treatment was associated with a reduction in participants' HbA1c following the use of a glucose monitoring system. The discrepancy may be due to the absence of an intervention in the present study that indicated a change in HbA1c, and the smaller sample size compared with those studies. Martínez et al.^37^ reported that low satisfaction with treatment was associated with higher HbA1c levels. The results obtained in this study agree with those reported by Toledo et al.^7^, although they did not evaluate the influence; just as in the present study, they found no relationship between these variables. This may be explained by the similarities in sociodemographic characteristics between the populations studied.

Among the limitations of this study is the sample size, since it is considered that the study could be more significant if the sample were larger. It is recommended to include other population groups for comparative purposes and to continue exploring these variables with other study designs (qualitative and mixed-method approaches).

## Conclusions

The results of this study show that adherence to pharmacological treatment influences glycemic control. Therefore, it is essential that healthcare personnel consider this evidence to improve decision- making in the clinical field and to guide the design, implementation, and reinforcement of educational interventions for adults with T2D.

In contrast, satisfaction with treatment was not related to either adherence or glycemic control, suggesting that individuals may report satisfaction with their treatment while still failing to adhere to pharmacological treatment, which in turn prevents glycemic control. Moreover, the findings indicate the need for further research on treatment satisfaction using other study designs, given the scarcity of evidence and the highly variable results.
